# How and why digital information interventions support patients and carers during hospital-to-home transitions: a protocol for a realist systematic review

**DOI:** 10.1136/bmjopen-2025-112464

**Published:** 2026-06-01

**Authors:** Evgenia Stepanova, Matthew Cooper, Lauren Lawson, Vicki Harris, Abbie Rance, Andy Husband, Fabiana Lorencatto, Wade Tovey, Elise Crayton, Clare L Tolley, Geoff Wong, Hamde Nazar

**Affiliations:** 1 Patient Safety Research Collaboration, National Institute for Health and Care Research, Newcastle Upon Tyne, UK; 2 Newcastle Health Research Partnership & Clinical Research Directorate, Newcastle Upon Tyne Hospitals NHS Foundation Trust, Newcastle upon Tyne, UK; 3 Connected Voice, Newcastle upon Tyne, UK; 4 School of Pharmacy, Newcastle University, Newcastle, UK; 5 University College London, London, UK; 6 North East Social Care Advisors CIC, Newcastle Upon Tyne, UK; 7 Centre for Behaviour Change, University College London, London, UK; 8 Nuffield Department of Primary Care Health Sciences, Oxford University, Oxford, UK

**Keywords:** Digital Technology, Health Services, Hospital to Home Transition, Hospitals, Public, Telemedicine

## Abstract

**Abstract:**

**Introduction:**

The transition from hospital to home is a vulnerable stage in the patient pathway. Patients and carers often report unmet information needs regarding diagnoses, medication changes, follow-up arrangements and escalation pathways during the post-hospital discharge period. Digital information interventions—such as electronic health records, patient portals or remote communication systems—have been proposed to improve discharge pathways. However, evidence on their impact is unproven. The aim of this review is to understand what works for whom, how, why and in what circumstances in relation to digital information interventions during the hospital-to-home journey.

**Methods and analysis:**

Pawson’s realist review approach will be used. The Preferred Reporting Items for Systematic Review and Meta-Analysis Protocols and Realist and Meta-narrative Evidence Syntheses: Evolving Standards quality and reporting standards will also be followed. The review will follow five steps: (1) Development of the initial programme theory; (2) evidence search; (3) selection and appraisal of data; (4) extraction and organisation of data and (5) data synthesis and analysis. The search will be conducted in MEDLINE (Ovid); Embase; PsycINFO; Web of Science and Cochrane Library and supplemented with citation tracking, grey literature, relevant organisational websites, programme evaluation reports and through consultation with stakeholders. The realist review will be an iterative process, and the initial realist programme theory will be tested (confirmed, refuted or refined) in response to the data searches and stakeholder discussions. Patient and public involvement and engagement will be embedded throughout the review. Patients, carers and health and care stakeholders will contribute to refining the initial programme theory, interpreting emerging programme theory and co-developing dissemination outputs to ensure findings remain grounded in lived realities.

**Ethics and dissemination:**

Ethical approval is not required for this review as it involves secondary analysis of published literature. The review will be conducted in accordance with principles of research integrity, transparency and responsible stakeholder involvement. Findings will inform the co-design of future digital discharge interventions and contribute to national priorities around digital transformation, safety and equity in transitional care. Dissemination will include conference presentations, a peer-reviewed journal article and accessible summaries co-developed with stakeholders to support equitable implementation and impact.

**STRENGTHS AND LIMITATIONS OF THIS STUDY**

- The realist review builds on extensive prior behavioural systems mapping and patient/public engagement and involvement, ensuring relevance to lived experiences of patients and carers and real-world practice challenges.
- Inclusion of quantitative, qualitative and grey literature enables examination of complex digital interventions but increases heterogeneity and reliance on variable reporting quality.
- Use of deductive, inductive and retroductive coding across multiple reviewers strengthens explanatory analysis, though realist inference necessarily involves interpretive judgement.
- Restricting inclusion to English-language studies may introduce language bias and limit the transferability of findings across international contexts.

## Introduction

 The transition from hospital to home is a fragile juncture in the patient care pathway, where lapses in information transfer and patient understanding may result in avoidable harm.^1 2^ Even when discharge appears ‘complete’, crucial information, such as diagnostic results, onward care provision or medication changes, often remain pending and are poorly communicated to the health and social care practitioners who provide care in the community.^3^,^6^

Patients and family carers consistently report unmet information needs immediately after discharge.^7^ In a study by Flacker *et al*, the association between the perceived provision of information to patients and the effective use of that information was explored. In a survey of older adults up to 3 days postdischarge, over half did not recall anyone discussing how they could care for themselves at home. This highlights how easily core messages can evaporate as people cross the hospital-to-community threshold.^8^ Medicines are a particular source of risk in the first few weeks following transfer: in a multicentre survey with follow-up at 2 to 3 weeks, over one-third of participants reported medicine-related problems, yet only 5% had spoken with a pharmacist, suggesting missed opportunities for timely, accessible support.^9^

Digital information interventions—ranging from electronic health record-integrated discharge tools and patient portals to support remote symptom reporting and pharmacist-facing handover systems—are frequently proposed to support the hospital–home divide.^10^ A recent systematic review of health technology-based discharge transition interventions found encouraging findings for patient experience and perceived discharge quality, but heterogeneous designs and outcomes limited firm conclusions on the impact on readmissions and emergency visits, pointing to a need for theory-driven evaluation.^4^ Case studies of national health information exchange further show that while patients can be given online access to parts of their records, interoperability and bidirectional communication constraints still blunt the impact for complex postdischarge care.^11 12^ Importantly, patient preferences and needs are not homogeneous. After discharge, while many people may choose telephone or online channels to seek advice for non-worrying symptoms, others value face-to-face contact. This highlights the need to address digital exclusion by ensuring that communication and follow-up options remain inclusive, adaptable and responsive to individuals’ differing levels of digital access and confidence.^13 14^

In 2025, the National Health Service in the UK released its 10-year vision for transforming the health service.^15^ There are ambitions to transition from analogue to digital services, to improve accessibility and efficiency and use AI-systems to enhance patient choice and personalise care. As part of this, the document describes a drive towards using a centralised app to support most patient communication and documentation.^15^

This review therefore focuses on digital information interventions targeted to patients and carers during the first few days (7–14) after hospital discharge to identify what works, for whom, in which contexts, through which mechanisms and towards what outcomes. We focus on how interventions address concrete needs in this window (eg, sense-making about diagnoses and results, medicines reconciliation and adverse effect management, when-to-seek-help advice and navigation back to services), how they support continuity with community practitioners (including availability and usefulness of discharge information at early follow-up) and how design choices—such as automation, interoperability and mechanisms for patient feedback and communication—mediate effects on safety, experience and utilisation. Findings are anticipated to support evidence-based design, implementation and adoption of digital communication approaches for patients and their carers over the hospital-to-home discharge journey.

## Methods

### Review aim

The aim of this realist review is to establish how digital information interventions support patients and carers during the hospital-to-home journey. Specifically, we will examine:

- Intervention—what digital information interventions and co-intervention strategies have been developed and tested.
- Context—what contextual factors influence the impacts of these interventions.
- Mechanism—how intervention components and accompanying strategies are theorised to trigger mechanisms of change.
- Outcomes—what intended and unintended outcomes are reported and how these relate to context and mechanisms.

Causal explanations developed will take the form of context–mechanism–outcome configurations (CMOCs).^16^

### Rationale for a realist review

Digital health interventions in transitional care are typically complex, adaptive and context-dependent.^17^ Their effects vary across populations, health systems and modes of delivery, necessitating the use of an approach that can unpack the complexity and account for variations in outcomes.^17^ Realist synthesis is designed to address these complexities by developing and refining a programme theory that explains what works, for whom, in what circumstances and why.^16 18^ This approach has been successfully applied to transitions of care and medicines-related interventions across transitions.^19^

### Review scope and questions

The research team has undertaken preparatory work within a prior funded project (National Institute for Health and Research Programme Development Grant, award number 205674). Patient and public involvement and engagement (PPIE) groups and groups of health and care stakeholders were established to investigate lived and professional experience of hospital-to-home discharge. Through iterative discussions and mapping exercises, the patient journey prior, at the point and immediately after the hospital discharge was considered in detail to understand the actors, actions and behaviours at play. Engagement occurred between July 2024 and May 2025 via in-person workshops, virtual meetings and collaborative review sessions. The findings allowed identification of key challenges that exist across this patient pathway, and PPIE discussions supported focusing the scope of further work on the form, nature and quality of communication in the short period (7–14 days) post-hospital discharge. This preparatory work ensured the focus of our research was relevant to health and care stakeholders, patients and carers. The realist review questions which emerged from the preparatory work were:

1. How, when and in what format should postdischarge information for patients and carers be communicated digitally so that they can understand and act on it without becoming information overloaded, while supporting appropriate and timely help-seeking?
2. How, when, for whom and why do digital interventions that clarify medicines and equipment use improve patient and carer confidence and safety at home?
3. In what ways, for whom, when, how and why do two-way communication features provide reassurance, enable sense-making and prevent ‘avoidable’ healthcare usage?
4. How do digital communication tools support clearer discharge pathways (who to contact, when and for what) and under what circumstances do the communication tools improve timely help-seeking behaviours?
5. In what contexts, for whom and through what mechanisms do digital communication tools improve consistency and effectiveness of communication across organisational boundaries (hospital, primary care, pharmacy, social care, carers and patients) and what outcomes result?

### Patient and public involvement and engagement

In line with realist methodology, patient, carer and stakeholder engagement will be integral to all stages of the review.^18 20^ Our PPIE group will include patients and carers with lived experience of hospital discharge and representatives from health and social care, voluntary sector and community organisations.

Participants will be purposefully selected to ensure representation of populations that are at increased risk of experiencing barriers during hospital-to-home transitions, particularly in relation to digital communication. These may include individuals from ethnic minority backgrounds, people with disabilities, those experiencing socioeconomic disadvantage and individuals with limited access to or confidence in digital technologies. Their inclusion is particularly important in this study, as digital information interventions may differentially affect groups depending on digital literacy, language, accessibility needs or access to devices and connectivity. Engaging these perspectives will enable the review to more critically examine mechanisms of digital exclusion, unintended consequences and contextual contingencies shaping intervention effectiveness.

Our PPIE group is planned to meet regularly throughout the project. Their roles will include:

- Advising on the scope of the review and refinement of the initial programme theory (IPT).
- Contributing to the interpretation of emerging CMOCs.
- Sense-checking the plausibility and resonance of findings with lived experience.
- Co-developing dissemination and practical outputs to share knowledge targeting a range of audiences (eg, plain-language summaries).

This iterative engagement will ensure that the developing theory remains grounded in lived realities and sensitive to equity considerations, consistent with guidance on involvement and realist review practice^21^ and UK Standards for Public Involvement.^22 23^

### Study methods

This review protocol is reported in accordance with the Preferred Reporting Items for Systematic Review and Meta-Analysis Protocols (PRISMA-P) statement.^24^ The review will be guided by Pawson’s iterative five-step process methodology.^25^ Reporting and conduct will follow the Realist and Meta-narrative Evidence Syntheses: Evolving Standards (RAMESES) to ensure rigour and transparency.^18^ Ethical approval is not required, as this study involves secondary analysis of published and publicly available data. Should additional realist interviews with stakeholders be undertaken, ethics approval will be sought separately.

#### Step 1: development of the IPT

Preparatory work—comprising engagement with existing literature,^1^,^510 12^ a mapping exercise and PPIE input—has already informed the development of a rudimentary version of IPT and potential CMOCs (phrased as ‘if… then… because…’ statements) (box 1).

Box 1 **Initial programme theory and potential context-mechanism-outcome configuration statements**

**Programme theory**

When people leave hospital, they (and their carers) enter a cognitively overloaded, information-sparse period where pending results, medicine changes, equipment use and ‘what-to-do-if’ advice are frequently unclear. Digital information interventions that (a) surface the right information at the right time, (b) deliver it in a clear, patient-actionable form, (c) provide tutorials, delivery tracking and supply alerts for safe equipment use, (d) offer processes for getting timely advice with primary and community health and care professionals, (e) offer digital check-ins with reassurance and emotional support signposting and (f) provide tiered escalation guidance on who to contact when problems arise, can reduce uncertainty and hazards. These interventions work when they fit patients’ access/preference constraints and health literacy, because they lower cognitive load, ensure people have the right tools and instructions at the right time, normalise recovery challenges, reduce anxiety, clarify escalation pathways, enable sense-making, and prompt help-seeking; leading to fewer errors, safer follow-up, and a better recovery experience.

**Candidate context–mechanism–outcome configurations**

Clear prompts for home care

***If*** *people forget much of their discharge self-care advice within the first few days, **then** bite-sized digital prompts that restate red-flag symptoms and what to do will increase appropriate help-seeking, **because** they reduce cognitive load and provide cues when they are needed*.

Medicines clarification+pharmacist link

***If*** *patients do not understand medicine changes and experience medication-related problems in the first 2–3 weeks, **then** providing a plain-language medicines summary plus a digital route to pharmacist support will reduce errors and problems, **because** it enables sense-making and rapid access to expertise*.

Modality fit to patient preference

***If*** *patients vary in their preferences and abilities to use digital tools (shaped by age, income, literacy, etc.), **then** offering multi-channel access (eg, phone, text, portal, app) and allowing choice will increase engagement, **because** it lowers barriers and aligns the intervention with user capability and comfort*.

Staggered timing of content

***If*** *patients are overloaded with information at discharge and forget important instructions, **then** digitally ‘dripped’ micro-content at day 2, 5, 10 will improve comprehension and adherence, **because** it matches information delivery to when it is most relevant and manageable*.

Carer-inclusive access

***If*** *carers who carry much of the burden of home care are given direct access to discharge information (including digital instructions, alerts, and action lists), **then** home care implementation will improve, **because** it empowers the person actually performing the tasks to act accurately and confidently*

Two-way feedback loops

***If*** *patients encounter problems or questions at home but find GP/community follow-up slow or hard to access, **then** interventions that provide quick, two-way communication (messaging or structured check-ins) will reduce unnecessary healthcare use, **because** they close the loop early and allow reassurance or adjustment before problems escalate*.

Digital handover to primary/community care

***If*** *community practitioners often receive incomplete discharge information, **then** standardised computerised discharge packets shared with both practitioners and patients will make early follow-up safer and more effective, **because** all parties work from the same, accurate, up-to-date information*.

Awareness on equipment readiness

***If*** patients are discharged without clarity on equipment use (eg, inhalers, dressings) or lack timely delivery of supplies, ***then*** digital discharge tools that provide tutorials, delivery tracking, and alerts to reorder supplies will reduce errors and anxiety, ***because*** they ensure patients and carers have the necessary knowledge at the right time.

Psychological reassurance

***If*** patients and carers experience anxiety, loneliness or uncertainty postdischarge, ***then*** digital check-ins (via text or app prompts with emotional support signposting) will reduce distress and build confidence, ***because*** they normalise recovery challenges and offer reassurance that help is available.

Follow-up care clarity across services

***If*** patients are unsure whether to contact hospital, GP, social worker, pharmacist, or emergency services when problems arise, ***then*** a clear, tiered digital escalation guide (with ‘who to call when’ decision trees) will improve timely and appropriate help-seeking, ***because*** it reduces hesitation and misdirected calls.

These initial CMOCs describe the possible causal processes at play during hospital-to-home transitions, the problems patients and carers encounter, and how and why digital interventions might improve outcomes. This preliminary theory draws on existing understanding of: (a) discharge processes and the actors and actions involved; (b) context-specific barriers and enablers affecting these processes and (c) evidence-informed approaches that may help to overcome barriers.

The IPT in box 1 will be further developed at the start of the realist review using the processes outlined below:

- scoping searches of academic and grey literature to identify relevant theories (eg, digital literacy, health communication, behaviour change, self-management);
- conceptual frameworks such as the Transitional Care Model^26^ and theories of health information engagement^12 17^;
- the expertise of the multidisciplinary research team; and
- input from PPIE representatives.

The IPT will act as a working model to guide evidence searching, selection and data extraction.

#### Step 2: evidence search

Systematic searches will be developed and revised as needed with an information specialist, incorporating stakeholder input and piloted before final execution.^27^ The IPT selected in step 1 will actively guide the selection and refinement of search terms, with further targeted searches undertaken to test, elaborate, or refute emerging contexts, mechanisms and outcomes as the review progresses.

It is important to identify data that provides insights into complexity, which will be reflected in the wide use of sources including, but not limited to, quantitative and qualitative data, peer-reviewed articles, commentaries and grey literature. We anticipate searches to be conducted in MEDLINE (Ovid); Embase; PsycINFO; Web of Science and Cochrane Library. Free-text and subject heading search terms will be chosen by the information specialist and discussed and refined (if needed) with the research team and stakeholders (see a online supplemental file for an example of MEDLINE search).

To capture non-indexed interventions, grey literature will be sought from OpenGrey, Trove, relevant organisational websites, programme evaluation reports, and through consultation with stakeholders. Additional strategies will include backward citation searching, forward citation (‘cited-by’) searching, kinship/sibling paper searching and snowballing.^14 25^

Searching will be conducted in iterative cycles and will continue until theoretical saturation is reached, defined as the point at which additional evidence no longer contributes substantively to the development or refinement of the programme theory. No date restrictions will be applied in order to capture the evolution of digital information interventions over time.

All retrieved results will be screened against predefined inclusion and exclusion criteria.

#### Inclusion criteria

Setting:

- Hospital-to-home transitions following discharge from any hospital setting.
- Interventions delivered at the point of or in the short period post-discharge (7–14 days).

Population

- Patients discharged from hospital.
- Patients aged 18 and over.
- Carers involved in discharge or postdischarge care.
- Healthcare professionals (eg, general practitioners, pharmacists, community nurses) if their involvement is part of the intervention’s communication loop.

Phenomenon of interest:

- Digital interventions designed to support patients and/or carers during hospital-to-home transitions. These may include wireless technology-based tools or platforms—such as mobile applications, text messaging systems, online portals or automated phone services—designed to support patients and/or carers during hospital-to-home transitions.

#### Exclusion criteria

- Interventions not linked to the transition from hospital to home.
- Non-digital interventions only.
- Non-healthcare settings.
- Digital interventions focused solely on administrative functions (eg, appointment booking) without a communication or information-support component.
- Information not published in English.

The data extraction template will include equity-relevant characteristics informed by the PROGRESS-Plus framework to enable exploration of differential impacts across population subgroups and contexts.

Databases will be searched from inception to May 2026.

#### Step 3: selection and appraisal of data

Search results will be screened against the inclusion criteria using a three-step process: title and abstract screening, full-text review and detailed assessment for theory contribution. Screening will be conducted independently in parallel by at least two members of the research team. A 10% random sample at each stage will be cross-checked to identify systematic inconsistencies. Disagreements will be resolved through discussion or consultation with a third reviewer to ensure consistency in study inclusion.

Consistent with realist methodology, inclusion will be guided by Pawson’s criteria of relevance and rigour.^25^ Relevance will be operationalised as the extent to which a document contains data capable of contributing to the development, refinement or testing of CMOCs. This includes evidence describing contextual conditions (eg, digital infrastructure, organisational readiness), mechanisms (eg, digital literacy, communication processes, patient engagement), implementation processes or outcomes associated with digital discharge interventions. Documents will be ranked according to their explanatory richness, distinguishing between conceptually rich studies that offer detailed insights into causal processes and studies providing more limited theoretical contribution.

Rigour will be assessed in terms of the credibility and trustworthiness of the methods used to generate the relevant data, in line with RAMESES quality standards.^28^ Rather than applying a traditional hierarchy of evidence, appraisal will consider whether the study methods are sufficiently robust to support the inferences drawn. This may include consideration of sampling strategy, data collection procedures, analytic transparency, appropriateness of study design and coherence between data and conclusions. Importantly, rigour will be assessed at the level of specific data fragments rather than entire studies. A study may therefore be included if particular findings are judged to be methodologically credible and theoretically informative, even if other aspects of the study are limited.

In line with realist principles, studies will be included if deemed ‘good enough’ by the review team for the purposes of theory development.^11 17 28^ Evidence of lesser methodological quality will not be automatically excluded if it offers valuable causal insights.

Given the iterative nature of realist synthesis, the review scope and selection criteria may be refined as the analysis progresses. Additional targeted searches may be undertaken where emerging programme theories require further testing or elaboration. Decisions regarding relevance, rigour and scope refinement will be documented to ensure transparency and auditability.

#### Step 4: extraction and organisation of data

Characteristics of the included documents such as bibliographic details, study design, population, intervention and cointervention characteristics will be extracted into an Excel spreadsheet by the review team. A data extraction form will be piloted on a sample of included studies and refined as necessary. Data extraction will be conducted independently by two reviewers for a subset of studies to ensure consistency. Documents then will be uploaded into NVivo, a computer programme used to support data analysis. Coding will proceed at three levels: deductive; inductive and retroductive.^11 17 29^ Deductive coding will be guided by the IPT, enabling data to be mapped against anticipated contexts, mechanisms and outcomes. Inductive coding will allow new themes, mechanisms or contextual factors to emerge from the evidence. Retroductive coding will infer underlying causal processes not directly observable.^17^ CMOCs will be developed by seeking explanatory connections across and within documents. Relevant sections of text will be extracted and coded according to how they contribute to testing or extending the programme theory.

In line with realist methodology, we will not apply a traditional risk of bias tool. Instead, appraisal will focus on relevance and rigour of specific data fragments, assessing whether the methods used to generate the data are credible and trustworthy for the purpose of theory building.

#### Step 5: data synthesis and analysis

Analysis and synthesis will take place iteratively during the review. This process will begin alongside document selection and appraisal (step 3) and continue through data extraction and organisation (step 4), guided by each data source’s potential to develop or test the programme theory.^17 28^

Data analysis will follow a realist logic of analysis. This involves examining the evidence to identify recurring outcome patterns (demi-regularities) and exploring how these can be explained through CMOCs. To do so, analytical strategies will include juxtaposition (comparing evidence from different sources to build a fuller explanation), reconciliation (examining contradictions to understand why similar contexts may produce different outcomes), adjudication (giving greater weight to more credible or trustworthy evidence) and consolidation (bringing insights together to develop middle-range theory).^11 17 28^

CMOCs will be developed by seeking explanatory data both within and across documents. Through this iterative process, evidence from diverse sources will be used to test and extend the IPT. The theory will continue to evolve as more data are analysed, offering a progressively more robust account of how, why and in what circumstances digital information interventions support patients and carers during hospital-to-home transitions.

Programme theory refinement will move continuously between the evidence, the IPT and stakeholder input. Emerging findings will be shared with the PPIE and stakeholder group to assess their plausibility, resonance and usefulness for practice and policy.^17 29^

### Reflexivity

This realist review is conducted within a broader funded programme focused on improving hospital-to-home transitions, including prior stakeholder and PPIE engagement that informed the IPT. The multidisciplinary team brings expertise in public health, pharmacy, behavioural science, digital health and transitional care. While this provides contextual insight, it may also shape assumptions about the value and feasibility of digital interventions.

The review is situated within a UK policy context emphasising digital transformation in healthcare. We recognise that such narratives may predispose interpretations toward digital optimism. To mitigate this, the IPT will be treated as provisional and subject to systematic testing and refinement, with active consideration of unintended consequences, including digital exclusion and inequities.

Reflexivity will be operationalised using a structured matrix informed by Rae and Green,^30^ guiding reflection across stages of theory development, data extraction, and analysis. Interpretive decisions, including adjudication of competing CMOCs, will be discussed collaboratively and documented. Ongoing PPIE involvement will provide an additional counterbalance to professional and policy-oriented perspectives.

## Ethics and dissemination

This project does not require ethical approval as it involves secondary analysis of published literature and does not include primary data collection involving human participants. The review will be conducted in accordance with principles of research integrity, transparency, and respect for stakeholders. These include:

- adherence to PRISMA-P and RAMESES reporting standards to ensure methodological transparency;
- systematic documentation of decisions regarding inclusion, appraisal, and theory development;
- reflexive consideration of interpretive bias throughout the synthesis process;
- responsible representation of evidence, including attention to equity and digital exclusion;
- meaningful and ongoing involvement of PPIE contributors, with recognition of their expertise and lived experience.

Ethical principles will guide engagement with the PPIE group. Recognising that hospital discharge experiences can be stressful or potentially distressing, particularly for individuals with vulnerabilities or limited digital access, we will adopt a trauma-informed approach to PPIE engagement consistent with established guidance. This includes creating psychologically safe and supportive environments for discussion, providing clear information about the purpose and use of contributions, respecting participants’ autonomy and boundaries, and explicitly acknowledging that individuals may choose not to discuss particular experiences.

Meetings will be structured to encourage trustworthiness, choice, collaboration, empowerment, and cultural sensitivity, reflecting core trauma-informed principles. Team researchers have been trained to recognise signs of distress and to respond supportively, and participants will be reminded that they can pause or withdraw at any time without consequence. PPIE contributors will be reimbursed in line with national guidance, and discussions and shared materials will be handled confidentially and respectfully.^31^

Findings will be disseminated through multiple knowledge mobilisation routes to maximise reach and impact. Academic dissemination will include submission of findings to a peer-reviewed journal and presentation at relevant national and international conferences.

User-centred outputs will be developed in accessible formats, including plain-language summaries and multimedia materials (eg, short videos or infographics), to ensure findings are accessible to patients, carers and the public.

Practice and policy engagement will include targeted briefings and workshops with clinicians and multidisciplinary discharge teams to support translation and local adaptation of findings.

## Discussion

The review is expected to generate an evidence-informed framework to guide the design, implementation, and evaluation of digital discharge interventions. Specifically, it will inform the co-design of a digital information intervention in later phases of the programme by identifying potential core components, contextual contingencies, and mechanisms that may influence patient and carer engagement during the hospital-to-home transition. The review is strengthened by its grounding in prior behavioural systems mapping and PPIE, ensuring relevance to lived experiences and real-world practice challenges. In addition, the inclusion of diverse evidence sources, including quantitative, qualitative and grey literature, will enable exploration of complex and context-dependent interventions.

However, several methodological limitations should be considered. As a realist synthesis, the review relies on interpretive judgement in identifying and refining CMOCs, which may introduce subjectivity despite efforts to ensure transparency and team-based consensus. The inclusion of heterogeneous evidence, including grey literature, may result in variable methodological quality, and while appraisal will focus on relevance and rigour, some inferences may be drawn from less robust data. The restriction to English-language publications may introduce language bias and limit the transferability of findings across international contexts.

Given these considerations, the findings of this review are intended to provide explanatory insights rather than definitive conclusions about effectiveness. The resulting programme theory will require further empirical testing and refinement in subsequent phases of the research.

Notwithstanding these limitations, the review has potential to inform national policy priorities around digital transformation, patient-centred care, and workforce sustainability, while critically addressing the risk that ‘digital by default’ approaches may exacerbate digital exclusion and health inequalities. By identifying not only whether digital interventions work, but how, for whom, and under what conditions, this review may support the development of inclusive, effective and sustainable solutions to improve hospital-to-home transitions.

## Supplementary material

10.1136/bmjopen-2025-112464 online supplemental file 1
