# Supplementary material for: How and why digital information interventions support patients and carers during hospital-to-home transitions: a protocol for a realist systematic review
Source: BMJ Open. 2026 Jun 1;16(6):e112464. doi: 10.1136/bmjopen-2025-112464 (PMC13239572; doi:10.1136/bmjopen-2025-112464)
Supplement: online supplemental file 1 [file bmjopen-16-6-s001.docx]

## MEDLINE

**Database:**
Ovid MEDLINE(R) <1996 to November Week 2 2025>

| **#** | **Query** | **Results from 13 Nov 2025** |
| --- | --- | --- |
| 1 | "patient* ".m_titl. | 1,719,130 |
| 2 | "caregiver* ".m_titl. | 26,080 |
| 3 | "carer* ".m_titl. | 4,435 |
| 4 | "family* ".m_titl. | 125,652 |
| 5 | "relative* ".m_titl. | 33,535 |
| 6 | Patients/ | 15,928 |
| 7 | Caregivers/ | 57,716 |
| 8 | Digital Health/ | 1,157 |
| 9 | Health Information Exchange/ | 1,204 |
| 10 | Internet/ | 87,130 |
| 11 | Internet-Based Intervention/ | 1,870 |
| 12 | Artificial Intelligence/ | 58,518 |
| 13 | Patient Portals/ | 1,010 |
| 14 | Telemedicine/ | 46,184 |
| 15 | Mobile Applications/ | 15,939 |
| 16 | ((web or website? or web-based or internet* or online or mobile* or computer* or patient* or tablet* or digital* or digiti* or electronic*) adj3 (application* or app? or portal* or platform* or hub? or dashboard*)).m_titl. | 12,653 |
| 17 | "digital health tool* ".m_titl. | 87 |
| 18 | "digital tool* ".m_titl. | 332 |
| 19 | "health information tool* ".m_titl. | 11 |
| 20 | Hospital to Home Transition/ | 73 |
| 21 | Patient Discharge/ | 37,854 |
| 22 | "hospital to home".m_titl. | 1,100 |
| 23 | "hospital discharge".m_titl. | 2,927 |
| 24 | ("postdischarge" or "post discharge").m_titl. | 2,026 |
| 25 | Transitional Care/ | 1,517 |
| 26 | (discharg* or post-hospital* or posthospital* or after-hospital*).m_titl. | 26,696 |
| 27 | 1 or 2 or 3 or 4 or 5 or 6 or 7 | 1,910,104 |
| 28 | 20 or 21 or 22 or 23 or 24 or 25 or 26 | 50,278 |
| 29 | 8 or 9 or 10 or 11 or 12 or 13 or 14 or 15 or 16 or 17 or 18 or 19 | 207,621 |
| 30 | 27 and 28 and 29 | 285 |
