# Supplementary material for: How and why digital information interventions support patients and carers during hospital-to-home transitions: a protocol for a realist systematic review
Source: BMJ Open. 2026 Jun 1;16(6):e112464. doi: 10.1136/bmjopen-2025-112464 (PMC13239572; doi:10.1136/bmjopen-2025-112464)
Supplement: Supplementary file 2 [file bmjopen-2025-112464.reviewer_comments.pdf]

PEER REVIEW HISTORY

BMJ Open publishes all reviews undertaken for accepted manuscripts. Reviewers are asked to complete a checklist review form and are provided with free text boxes to elaborate on their assessment. These free text comments are reproduced below.

ARTICLE DETAILS

Title (Provisional)

How and why digital information interventions support patients and carers during hospital-to-home transitions: a protocol for a realist systematic review.

Authors

Stepanova, Evgenia; Cooper, Matthew; Lawson, Lauren; Harris, Vicki; Rance, Abbie; Husband, Andy; Lorencatto, Fabiana; Tovey, Wade; Crayton, Elise; Tolley, Clare L; Wong, Geoff; Nazar, Hamde

---

VERSION 1 - REVIEW

**Reviewer**	**1**
**Name**	**Evans, Catrin**
**Affiliation**	**University of Nottingham, School of Health Sciences,**
**Date**	**19-Dec-2025**
**COI**	**None**

Thank you for the opportunity to review this excellent protocol. It sets out an enquiry that is rigorous and novel. I have very few substantive comments. I offer a few suggestions below for the authorship team to consider.

Title

• This could indicate a realist focus more clearly, e.g. “Investigate the evidence” sounds a bit like a scoping review

Inclusion Criteria

• I wonder if the authors should consider specifying a date range? Given the changes in communication and digital literacy that have taken place since Covid, it may be helpful to limit empirical studies to a recent date range

• Will languages other than English be included?

Data Extraction

• It may help the review team to maintain an equity focus within the review if part of the data extraction template utilises established equity frameworks for some of the population or study characteristics (e.g. Progress-Plus)

Assessment of Rigor and Relevance

• Can the authors provide more detail regarding how these concepts will be operationalised and assessed?

Reflexivity

• In line with good practice in review methods, can the team include a reflexive statement around the team and the project and how reflexivity will be addressed going forwards

---

**Reviewer**	**2**
**Name**	**Elmer, Shandell**
**Affiliation**	**University of Tasmania**
**Date**	**29-Jan-2026**
**COI**	**None**

Thank you for outlining the plan for your research in this important area. Transitions of care are high risk for patients and their family/carer as the burden for understanding what to do next is transferred to those often least prepared to know and remember. The initial programme theory and potential CMOCs developed by the research team demonstrate in depth insight and understanding of the realities of these situations.

I offer the following suggestions for your consideration:

Abstract

Line 40: Ethics and dissemination – I agree that ethics approval is not required, however, please consider making mention of the ethical principles that will be followed – in the abstract this can be a short mention, but in the body of the paper, how these ethical principles will be enacted could be described.

Line 47: There is no mention of limitations in the abstract. I note also that the strengths and limitation section is not included in the body of the paper.

Review scope and questions

Line 41: Minor point, please write in full Patient and Public Involvement and Engagement (involvement is missing here)

Line 54: For ease of reference, it might be advisable to number the realist review questions.

Line 54 RQ1: There may be many reasons that patient and their family carers are unable to act on information, including but not limited to information overload. To enable more of an exploratory approach, I suggest removing this part of the question.

Line 4 RQ3: I think I understand the intention here is to prevent 'avoidable' healthcare, not healthcare that may be appropriate or warranted. Is this correct? Please consider if the wording of this RQ needs to be revised for clarity.

Line 6 RQ4: Please consider if this RQ can be reworded for clarity, particularly the second part of the question – be more specific about what "this" is referring to. Is the emphasis on the communication tools (under what circumstances do the communication tools…) or the discharge pathways (under what circumstances does understanding the discharge pathways…).

Line 11 RQ5: This RQ does not have the same realist qualities as the other questions. Consider if this RQ could be rephrased to reflect the realist endeavour.

Patient and Public Involvement and Engagement (PPIE)

Line 22: It is laudable to reach out to groups that do not usually have the opportunity to participate in research, however, this section could be strengthened by providing a rationale for their inclusion within this study in particular.

Study methods

Line 47: Please check reference used here [26] – this reference Moher et al, (2015) does not mention Pawson. The earlier Pawson references [11] and [17] might be more appropriate although I note the 2006 text has 6 steps in the protocol, whereas the earlier (2005) has 5.

Line 50: As mentioned previously, while ethical approval is not required, this section could be strengthened by describing the ethical principles that will be applied to working with your PPIE group. I note reference to the UK Standards for Public Involvement, however, this appears to be more guidance for working with patients and the public, rather than application of ethical principles. Consider also the inclusion of a trauma informed approach, particularly as you have identified the inclusion of people with lived experience and with the least exposure to involvement in research, and most at risk of the vulnerabilities around care transitions.

Line 51: Stakeholders are referred to here – is this inclusive of the PPIE group? If so, perhaps clarify this.

Step 1

Line 22: Second dot point – please check relevance of reference [17] here

Step 2

Line 44: Check relevance of reference [14] here

Inclusion criteria

Line 58: Please clarify if the inclusion criterion is Adult Patients only

Step 3

Line 30: Please check relevance of reference [26] here

Line 39: Please check references – should these be 17 and 19 instead of 16 and 18. Is it possible that your reference list could be out of synch? Please check.

Step 4

Line 48: Please check reference [16] here

Line 52: Please check reference here – should this be [22]?

Step 5 – please check referencing throughout this section

Line 29: I suggest that ethics does not need to be discussed here again.

Line 55: The strengths and limitations are mentioned in the abstract but are not included in the body of the paper. Please consider including this section here, ensuring that the limitations of the study are described.

VERSION 1 - AUTHOR RESPONSE

Reviewer: 1<br>Dr. Catrin Evans, University of Nottingham<br>Comments to the Author:<br>Thank you for the opportunity to review this excellent protocol. It sets out an enquiry that is rigorous and novel. I have very few substantive comments. I offer a few suggestions below for the authorship team to consider.	Many thanks for the positive feedback.
Title<br>• This could indicate a realist focus more clearly, e.g. "Investigate the evidence" sounds a bit like a scoping review	The title has been revised to demonstrate a more explanatory-based, theory-building realist review.
Inclusion Criteria<br>• I wonder if the authors should consider specifying a date range? Given the changes in communication and digital literacy that have taken place since Covid, it may be helpful to limit empirical studies to a recent date range<br>• Will languages other than English be included?	Many thanks for this. The Inclusion and exclusion criteria have been narrowed down to English-language only publications. With regards to the date range, thank you for this helpful suggestion. We agree that the COVID-19 pandemic has significantly influenced digital communication practices and digital literacy in healthcare. However, as this study adopts a realist review methodology, the aim is not only to identify recent effectiveness evidence but to develop and refine programme theory by understanding how, why, and under what circumstances digital information

	interventions work. Earlier studies can provide important explanatory insights into mechanisms (e.g. communication processes, engagement, trust, and access barriers) that remain relevant across time and contexts, even if the technological landscape evolves.<br>Therefore, we have not restricted the search by publication date in order to capture the evolution of digital discharge communication and identify transferable mechanisms. To ensure contemporary relevance, we will interpret findings in light of current healthcare practice and highlight where mechanisms may operate differently in post-COVID contexts.
Data Extraction<br>• It may help the review team to maintain an equity focus within the review if part of the data extraction template utilises established equity frameworks for some of the population or study characteristics (e.g. Progress-Plus)	Thank you for this helpful suggestion. We agree that maintaining an explicit equity focus is important, particularly given the risk of digital exclusion associated with digital health interventions. We will incorporate equity-relevant characteristics into the data extraction template, drawing on the PROGRESS-Plus framework to guide the identification of population and contextual factors where reported.
Assessment of Rigor and Relevance<br>• Can the authors provide more detail regarding how these concepts will be operationalised and assessed?	Thank you for this helpful comment. We have expanded the section on assessment of relevance and rigour to clarify how these concepts will be operationalised in practice, including explicit criteria for assessing explanatory contribution, methodological credibility, fragment-level appraisal, and documentation of decisions in line with RAMESES standards.
Reflexivity<br>• In line with good practice in review methods, can the team include a reflexive statement around the team and the project and how reflexivity will be addressed going forwards	Thank you for this valuable suggestion. We have now included a reflexive statement outlining the multidisciplinary positioning of the team and describing how reflexivity will be operationalised throughout the review using a structured matrix informed by Rae and Green (2016).
Reviewer: 2<br>Dr. Shandell Elmer, University of Tasmania<br>Comments to the Author:<br>Thank you for outlining the plan for your research in this important area. Transitions of care are high risk for patients and their family/carer as the burden for understanding	Many thanks for your thorough feedback and suggestions for improvement.

what to do next is transferred to those often least prepared to know and remember. The initial programme theory and potential CMOCs developed by the research team demonstrate in depth insight and understanding of the realities of these situations.<br>I offer the following suggestions for your consideration	
Abstract<br>Line 40: Ethics and dissemination – I agree that ethics approval is not required, however, please consider making mention of the ethical principles that will be followed – in the abstract this can be a short mention, but in the body of the paper, how these ethical principles will be enacted could be described.	Thank you for this helpful suggestion. While formal ethical approval is not required for this review, we have now clarified the ethical principles guiding the conduct of the study in both the abstract and the main manuscript. These include adherence to research integrity and transparency standards, reflexive practice, responsible representation of evidence, and meaningful PPIE involvement.
Line 47: There is no mention of limitations in the abstract. I note also that the strengths and limitation section is not included in the body of the paper.	Many thanks, the strengths and limitations have been added.
Review scope and questions<br>Line 41: Minor point, please write in full Patient and Public Involvement and Engagement (involvement is missing here)	Thank you, amendment has been added
Line 54: For ease of reference, it might be advisable to number the realist review questions.	Thank you, amended.
Line 54 RQ1: There may be many reasons that patient and their family carers are unable to act on information, including but not limited to information overload. To enable more of an exploratory approach, I suggest removing this part of the question.	Thank you for this helpful clarification. The intention is indeed to support appropriate and timely help-seeking rather than reduce warranted healthcare use. We have revised the wording of the research question to make this distinction clearer.
Line 4 RQ3: I think I understand the intention here is to prevent 'avoidable' healthcare, not healthcare that may be appropriate or warranted. Is this correct? Please consider if the wording of this RQ needs to be revised for clarity.	Many thanks for suggesting clarification. Yes, this is absolutely correct.
Line 6 RQ4: Please consider if this RQ can be reworded for clarity, particularly the second part of the question – be more specific about what "this" is referring to. Is the emphasis on the communication tools (under what circumstances do the communication tools…)	Thank you for suggesting this. We have amended it accordingly

or the discharge pathways (under what circumstances does understanding the discharge pathways…).	
Line 11 RQ5: This RQ does not have the same realist qualities as the other questions. Consider if this RQ could be rephrased to reflect the realist endeavour.	Thank you for this helpful observation. We agree that the original wording did not fully reflect the realist orientation of the review. The research question has been revised to explicitly incorporate context, mechanisms, and outcomes, in line with the realist approach.
Patient and Public Involvement and Engagement (PPIE)<br>Line 22: It is laudable to reach out to groups that do not usually have the opportunity to participate in research, however, this section could be strengthened by providing a rationale for their inclusion within this study in particular.	Thank you for this helpful suggestion. We have strengthened the PPIE section to clarify the rationale for including underserved groups in the context of this study. Specifically, we now explain how digital discharge interventions may differentially affect populations at risk of digital exclusion, and how their involvement is essential to understanding contextual and equity-related mechanisms.
Study methods<br>Line 47: Please check reference used here [26] – this reference Moher et al, (2015) does not mention Pawson. The earlier Pawson references [11] and [17] might be more appropriate although I note the 2006 text has 6 steps in the protocol, whereas the earlier (2005) has 5.	Many thanks for pointing this out. We have revised the inconsistencies in references.
Line 50: As mentioned previously, while ethical approval is not required, this section could be strengthened by describing the ethical principles that will be applied to working with your PPIE group. I note reference to the UK Standards for Public Involvement, however, this appears to be more guidance for working with patients and the public, rather than application of ethical principles. Consider also the inclusion of a trauma informed approach, particularly as you have identified the inclusion of people with lived experience and with the least exposure to involvement in research, and most at risk of the vulnerabilities around care transitions.	Thank you for this important suggestion. We have strengthened the Ethics and Dissemination section to clarify the ethical principles guiding our work with the PPIE group. We will adopt a trauma-informed approach to PPIE engagement, consistent with established practice guidance (Imperial College, 2024), emphasising trust, choice, collaboration, and cultural sensitivity
Line 51: Stakeholders are referred to here – is this inclusive of the PPIE group? If so, perhaps clarify this.<br>Step 1<br>Line 22: Second dot point – please check relevance of reference [17] here	Thanks for this clarification. Please note that under stakeholders the manuscript refers to 'care stakeholders' which have been mentioned in Methods and analysis section.

Step 2<br>Line 44: Check relevance of reference [14] here	Thank you, the references and in-text citations have been revised accordingly.
Inclusion criteria<br>Line 58: Please clarify if the inclusion criterion is Adult Patients only	Many thanks for this helpful comment. It has been clarified.
Step 3<br>Line 30: Please check relevance of reference [26] here	Thank you, the references and in-text citations have been revised accordingly.
Line 39: Please check references – should these be 17 and 19 instead of 16 and 18. Is it possible that your reference list could be out of synch? Please check.	Thank you, the references and in-text citations have been revised accordingly.
Step 4<br>Line 48: Please check reference [16] here	Thank you, the references and in-text citations have been revised accordingly.
Line 52: Please check reference here – should this be [22]?	Thank you, the references and in-text citations have been revised accordingly.
Step 5 – please check referencing throughout this section	Thank you, the references and in-text citations have been revised accordingly.
Line 29: I suggest that ethics does not need to be discussed here again.	
Line 55: The strengths and limitations are mentioned in the abstract but are not included in the body of the paper. Please consider including this section here, ensuring that the limitations of the study are described.	Many thanks for highlighting this. In accordance with the journal’s formatting requirements, the strengths and limitations are included below the abstract.
